# High tibial osteotomy effectively restores motor function during daily activities in patients with knee osteoarthritis and varus deformity

**DOI:** 10.1002/jeo2.70410

**Published:** 2025-09-15

**Authors:** Giordano Valente, Giulia Grenno, Giacomo Dal Fabbro, Alberto Grassi, Alberto Leardini, Lisa Berti, Stefano Zaffagnini, Fulvia Taddei

**Affiliations:** ^1^ Bioengineering and Computing Laboratory IRCCS Istituto Ortopedico Rizzoli Bologna Italy; ^2^ 2nd Orthopedics and Trauma Unit IRCCS Istituto Ortopedico Rizzoli Bologna Italy; ^3^ Department of Biomedical and Neuromotor Sciences University of Bologna Bologna Italy; ^4^ Movement Analysis Laboratory IRCCS Istituto Ortopedico Rizzoli Bologna Italy; ^5^ Physical Medicine and Rehabilitation IRCCS Istituto Ortopedico Rizzoli Bologna Italy

**Keywords:** gait analysis, high tibial osteotomy, osteoarthritis, varus knee

## Abstract

**Purpose:**

This study evaluated the effects of high tibial osteotomy (HTO) on spatio‐temporal parameters, kinematics and kinetics during walking, stair ascent and descent, in patients with medial knee osteoarthritis and varus malalignment, by using a prospective randomized case‐control design, which compares the effects of HTO with a non‐surgical conservative treatment.

**Methods:**

A total of 49 patients with medial knee osteoarthritis and varus malalignment were enroled in a prospective randomized case‐control study. Patients were randomly assigned to the Surgical Group (*n* = 25), which underwent open‐wedge HTO, or the Conservative Group (*n* = 24), which followed non‐surgical conservative treatment. An additional 20 healthy subjects were included as controls. Radiographs in double‐leg stance and gait analysis during the motor activities were conducted at baseline and follow‐up. Statistical comparisons of spatio‐temporal parameters, joint rotations and joint moments were performed to assess the effects of surgery on motor function.

**Results:**

In the Surgical Group, HTO significantly corrected the tibiofemoral angle (from 8.3 ± 3.3° to 0.9 ± 2.4°, *p* < 0.001), restoring values comparable to healthy controls. Knee and ankle adduction were fully restored in all motor tasks, with significant improvements in at least 60% of the movement cycle (*p* < 0.05). Knee adduction and rotation moments were significantly reduced, with some patients even showing lower‐than‐normal knee adduction moments during walking, suggesting possible overcorrection. However, pelvic obliquity and ankle flexion remained altered, and no significant changes were observed in walking speed or stride length. The Conservative Group showed no improvements at follow‐up.

**Conclusions:**

HTO effectively restores knee alignment and major motor function parameters during walking and stair tasks, whereas non‐surgical conservative treatments do not lead to any improvement. However, some residual motor function deviations persist after surgery, suggesting that certain biomechanical adaptations may remain after surgical correction.

**Level of Evidence:**

Level I, randomized controlled trial.

**Abbreviations**
HTO: high tibial osteotomy
KAM: knee adduction moment
KFM: knee flexion moment
KL: Kellgren–Lawrence
KRM: knee rotation moment
MPTA: mechanical medial proximal tibial angle
OA: osteoarthritis
TFA: tibiofemoral alignment
VAS: visual analogue scale

## INTRODUCTION

People affected by knee osteoarthritis (OA) often experience altered motor function that leads to limitations in daily activities and reduced quality of life. In this context, knee varus malalignment represents a major risk factor for the progression of medial compartment OA and cartilage degeneration, as it increases mechanical loading on the medial compartment, accelerating joint deterioration [1, 8, 24, 25]. Gait analysis studies have shown that patients with knee varus deformity exhibit increased knee adduction moment (KAM) and knee rotation moment (KRM), decreased knee flexion moment (KFM), as well as altered lower limb kinematics during walking and stair tasks [8, 10, 11, 27, 28]. In addition, recent research found that varus severity has a greater influence on the altered motor function parameters than OA grade and previous meniscectomy, with larger varus angles being associated with significantly greater alterations in biomechanical parameters [29].

High tibial osteotomy (HTO) is a well‐established joint‐preserving surgical procedure aimed at realigning the lower limb, reducing medial compartment loading and slowing OA progression [12, 17]. After HTO, patients experience increased walking speed and stride length, as well as normalized knee flexion during stance [15, 18], although some studies reported no significant changes in spatio‐temporal parameters [2, 20]. HTO significantly reduces KAM and lateral thrust [2, 7, 15, 18, 33], also correcting compensatory gait adaptations such as excessive frontal plane trunk sway [28]. However, while KAM reduction is a primary goal of HTO, its relationship with functional gait recovery remains complex [5]. Previous research showed that, although improvements in KAM are evident after HTO, transverse and sagittal plane kinetics may not fully normalize. Specifically, while KRM is significantly reduced after surgery [2, 16, 33], KFM remained unaltered or not fully restored, indicating a persistent functional deficit in the sagittal plane [2, 6, 16, 33], although these findings are controversial [18, 19]. Additional gait deviations persist after surgery, including reduced sagittal plane knee range of motion and increased stance duration compared to healthy controls [33]. In addition, HTO correction angles between 5° and 10° were found to influence tibiofemoral kinematics at knee flexion of 80° and more [6]. Since functional recovery depends on the interplay of multi‐planar joint loading, a comprehensive understanding of these biomechanical adaptations is crucial for optimizing post‐operative rehabilitation strategies.

Despite these known effects, the impact of HTO on motor function remains underexplored, especially through instrumental gait analysis. While HTO can effectively reduce pain and improve knee function compared to non‐surgical treatments [22, 26], its impact on gait parameters is not clear. Particularly, no prospective randomized case–control studies have systematically assessed the relationship between HTO and motor function outcomes during daily living activities.

Therefore, the aim of this study was to evaluate whether and to what extent HTO effectively restores motor function, assessed through spatio‐temporal parameters, kinematics and kinetics during walking, stair ascent and stair descent in patients with medial knee OA and knee varus deformity. We hypothesized that patients undergoing HTO would demonstrate greater improvements in motor function compared to those receiving non‐surgical conservative treatment, in a prospective case‐control clinical study.

## METHODS

### Study design and patient characteristics

Table S1 with medial knee OA and varus malalignment were initially recruited. Inclusion criteria were: age between 40 and 65 years, presence of medial knee pain, radiographic OA severity ≤ 3 according to Kellgren–Lawrence (KL) classification, and varus malalignment > 4°. The recruited patients included KL Grade 2 and 3 [29]. Exclusion criteria were: joint infection, inflammatory chronic disease, previous major surgical procedure of the indexed knee, and BMI > 30 kg/m^2^ [29]. At follow‐up, three patients dropped out, leaving a final cohort of 49 patients. These patients were randomly subdivided into two groups, according to a partially randomized patient preference design [4, 5]. The Surgical Group (n = 25) underwent an open‐wedge HTO technique, with hardware removal occurring approximately 12 months after surgery and before the assessment at follow‐up. The Conservative Group (*n* = 24) followed a non‐surgical management plan, consisting of weight control and standard muscular reinforcement that included weekly isometric exercises for the quadriceps, swimming and cycling. Furthermore, the use of an insole with a 2 mm raised external profile with a valgus effect was prescribed. The compliance of patients and the correct execution of the programme were periodically reviewed through in‐person follow‐up examinations in the physiotherapy department of the authors' institution. The patients of the Conservative Group did not receive knee injections during the period between baseline and follow‐up. The visual analogue scale (VAS) single score for both groups at baseline and follow‐up was chosen as a patient‐reported outcome measure for pain intensity. No significant differences in the patients' characteristics were observed between the Surgical and Conservative groups at baseline (Mann–Whitney *U* tests, *α* = 0.05, Table S1). A priori power analysis that focused on cartilage T2 variations following HTO (*t* test, expected T2 difference 3.5 ms, standard deviation 4.5 ms, significance level 0.05, power 0.8), indicated a required sample size of 44 patients. Regarding this specific study on gait biomechanics, post hoc power analysis on the difference between two dependent means (matched pairs pre‐ and post‐treatment) for the primary outcome variable peak KAM, indicated a statistical power of 1 with our sample size of 25 patients (Surgical Group). In addition, 20 healthy subjects without knee OA or varus deformity were included in this study as healthy controls (Healthy Group, *n* = 20). The Healthy Group was derived from an existing normative dataset acquired under the same gait analysis protocol. No radiographic imaging was available for these participants, and the tibiofemoral alignment (TFA) was calculated from marker‐based posture during standing trials. Group characteristics are reported in Table 1.

**Table 1:** Subjects' characteristics (baseline, follow‐up and healthy) for the Surgical and Conservative groups.

Surgical group							
	Baseline	Follow‐up	Healthy	Baseline vs. follow‐up	Baseline vs. healthy	Follow‐up vs. healthy	Effect size – baseline vs. follow‐up
	Mean (std)	Mean (std)	Mean (std)	*p*	*p*	*p*	Cohen's *d*
Number of participants	25	25	20	‐	‐	‐	‐
Age (years)	54.3 (6.8)	55.8 (6.7)	28.4 (5.1)	0.758	**<0.001**	**<0.001**	−0.22
Gender (F/M)	7F/18M	7F/18M	10F/10M	‐	‐	‐	‐
Body mass index (kg/cm^2^)	26.9 (4.0)	28.0 (4.2)	22.2 (2.9)	0.781	**<0.001**	**<0.001**	−0.27
Tibiofemoral angle (deg)	8.3 (3.3)	0.9 (2.4)	−0.3 (3.0)	**<0.001**	**<0.001**	0.750	**2.51**
Joint line convergence angle (deg)	3.1 (3.6)	2.4 (3.5)	‐	0.050	‐	‐	0.20
Medial proximal tibial angle (deg)	84.5 (2.8)	91.8 (3.2)	‐	**<0.001**	‐	‐	−**2.43**
VAS	6.0 (2.1)	1.6 (1.9)	‐	**<0.001**	‐	‐	**2.20**

Conservative group							
	Baseline	Follow‐up	Healthy	Baseline vs. follow‐up	Baseline vs. healthy	Follow‐up vs. healthy	Effect size *–* baseline vs. follow‐up
	Mean (std)	Mean (std)	Mean (std)	*p*	*p*	*p*	Cohen's *d*
Number of participants	24	24	20	‐	‐	‐	‐
Age (years)	51.6 (10.0)	53.0 (9.9)	28.4 (5.1)	0.859	**<0.001**	**<0.001**	−0.14
Gender (F/M)	2F/22M	2F/22M	10F/10M	‐	‐	‐	‐
Body mass index (kg/cm^2^)	25.6 (3.1)	25.9 (3.4)	22.2 (2.9)	0.993	**0.003**	**0.002**	−0.09
Tibiofemoral angle (deg)	7.7 (4.0)	7.6 (4.4)	−0.3 (3)	0.973	**<0.001**	**<0.001**	0.02
Joint line convergence angle (deg)	1.5 (2.5)	1.9 (2.6)	‐	**0.005**	‐	‐	−0.16
Medial proximal tibial angle (deg)	84.0 (2.6)	84.1 (2.6)	‐	0.517	‐	‐	−0.04
VAS	4.0 (2.1)	2.9 (2.3)	‐	0.063	‐	‐	0.50

*Note*: Bold *p* values indicate statistically significant differences.

Abbreviation: VAS, visual analogue scale.

### Surgical technique

All surgeries were performed by a well‐experienced knee surgeon (i.e., senior Author SZ), according to the same surgical procedure. The preoperative planning was executed on the full‐length radiographs in a double stance. The correction was planned to obtain a post‐operative mechanical TFA as near as possible to neutral (180–182°), without exceeding 95° of mechanical medial proximal tibial angle (MPTA). A 10 cm longitudinal skin incision was made at the proximal anteromedial aspect of the tibia. The medial proximal tibia was exposed by elevating the insertion of the pes anserinus and the hamstrings tendons, and by slightly releasing the superficial layer of the medial collateral ligament. After checking the inclination and position of the cut through a 2.5 mm diameter K‐wire, a biplanar opening wedge osteotomy was performed. The gap size was checked with a graduated spreader to obtain the target correction, and the osteotomy was then secured with a plate and screws (TOMOFIX osteotomy system, DePuy Synthes). The osteotomy opening gap was then filled with an allograft bone wedge. Following the surgery, the knee was placed in an extension brace for 4 weeks, removable during the day for the range of motion exercises, which were allowed from the second day after surgery. Following an initial touch‐weight‐bearing period of 4 weeks, progressive weight bearing as tolerated was allowed. All the patients followed the same post‐operative rehabilitation protocol supervised at the physiotherapy department of the authors' institution.

### Radiographic and gait analysis data

Radiographic and gait analysis data of the patients were collected at two time points: baseline (pre‐treatment), corresponding to the time of patient recruitment, and follow‐up (post‐treatment), occurring on average 19.5 ± 6.6 months after intervention for the Surgical Group and 16.3 ± 5.6 months for the Conservative Group. Gait analysis for the Healthy Group was performed at a single time point.

Long‐leg weight‐bearing radiographs in a double‐leg stance were acquired to measure the mechanical TFA, which was defined as the angle between the femoral mechanical axis, connecting the hip and knee joint centres, and the tibial mechanical axis, connecting the knee and ankle joint centres (Table 1).

**Figure 1:**
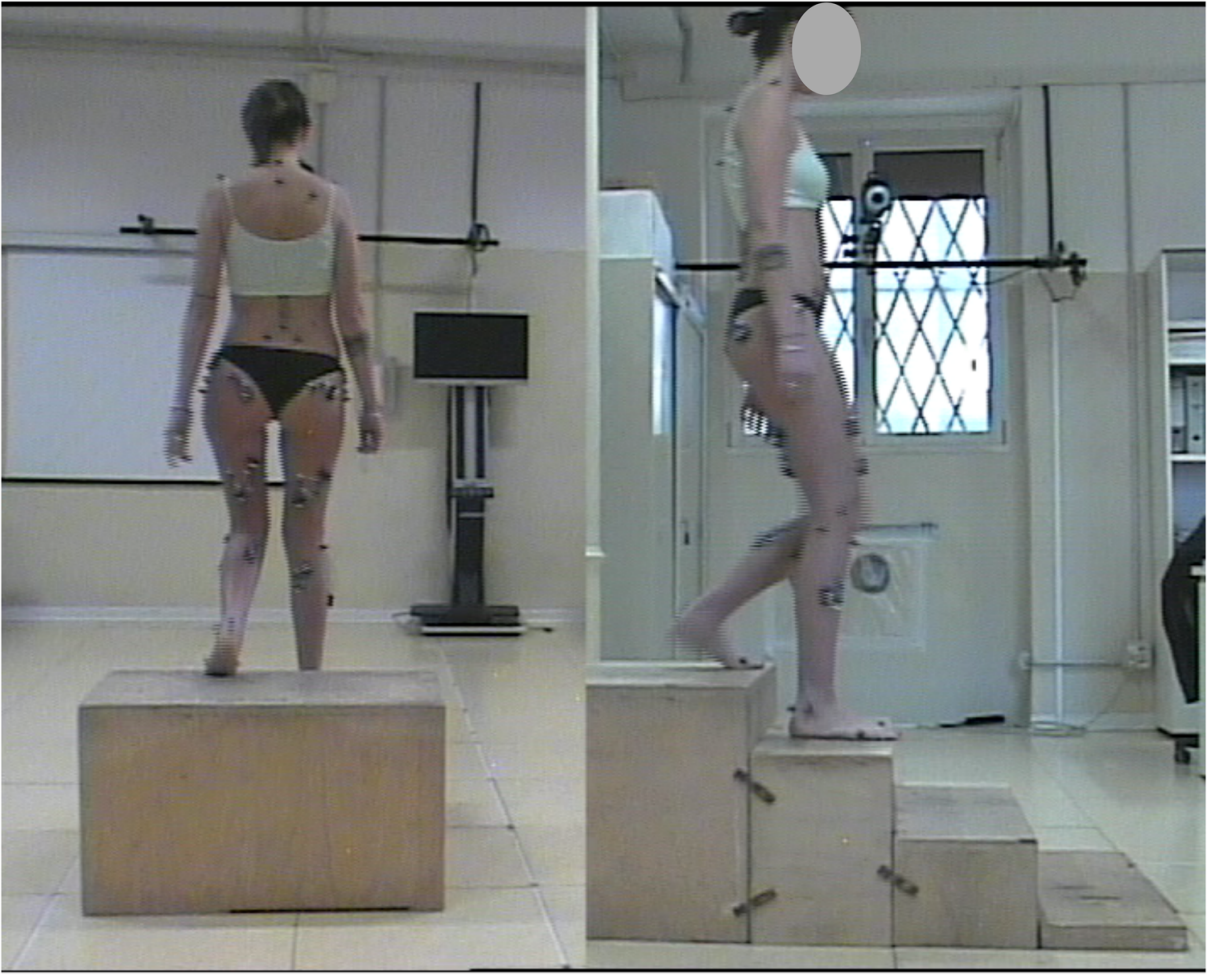
Setup of data collection for motion capture during a stair descent session of a patient.

Motion capture data, including 3D marker trajectories and ground reaction forces, were acquired during walking, stair ascending and stair descending for five repetitions each. The patients were first instrumented with 30 reflective markers on lower limbs, pelvis and trunk according to the established IOR‐gait and trunk marker sets and protocols [13, 14]. Then, motion capture data were simultaneously collected using an 8‐camera motion capture system at 100 Hz (Bonita, Vicon Motion System Ltd) and two embedded force plates at 2000 Hz (Kistler). The setup of gait analysis data collection is shown in Figure 1, and additional details have been described previously [29, 30].

### Data processing and statistical analysis

To assess the effects of HTO on motor function, statistical comparisons were performed between baseline and follow‐up, as well as between patients and healthy controls, for both the Surgical and Conservative groups.

Subject characteristics and spatio‐temporal parameters were compared among the three conditions (baseline, follow‐up and healthy) separately for the Surgical and Conservative groups. A Kruskal–Wallis test was used for group comparisons, and in cases of significant differences (*α* = 0.05), post hoc pairwise comparisons were conducted using Tukey–Kramer adjustments. In addition, Cohen's *d* effect sizes were calculated to complement statistical comparisons between baseline and follow‐up parameters for the Surgical and Conservative groups.

Kinematics and kinetics data, including joint rotations, ground reaction forces and joint moments, were first normalized to the percentage of the movement cycles and expressed as mean and standard deviation. Then, statistical comparisons of kinematics and kinetics parameters among the three conditions (baseline, follow‐up and healthy) during the three motor activities were performed separately for the Surgical and Conservative groups using statistical parametric mapping (SPM) [21], with the SPM1D package (v0.4, www.spm1d.org [23]) implemented in MATLAB. Specifically, a non‐parametric repeated measures ANOVA was used to detect overall differences, followed by non‐parametric two‐tailed paired t‐tests for post‐hoc pairwise comparisons when significant effects were found (*α* = 0.05). Differences were considered clinically relevant if statistically significant effects were observed over at least a consecutive 4% of the movement cycle [31, 32]. Only the parameters that showed significant differences were reported in the following analysis. In addition, to assess the effects of the two treatments, statistical comparisons between the Surgical and Conservative groups were performed at both baseline and follow‐up. As for the previous analysis, non‐parametric SPM *t* tests were applied to the kinematic and kinetic parameters during walking, stair ascent and stair descent, with the aim to verify whether inter‐group differences emerged post‐treatment despite similar biomechanical profiles at baseline. This additional analysis is reported in the Supporting Information [Link], [Link], [Link], [Link].

## RESULTS

### Subject characteristics and spatio‐temporal parameters

In the Surgical Group, the TFA significantly decreased from 8.3 ± 3.3° at baseline to 0.9 ± 2.4° at follow‐up (*p* < 0.001), reaching values comparable to those of the Healthy Group (−0.3 ± 3.0°, p = 0.750). In contrast, the Conservative Group showed no significant changes in TFA between baseline (7.7 ± 4.0°) and follow‐up (7.6 ± 4.4°) (*p* = 0.973), while both values remained significantly different from those of the Healthy Group (*p* < 0.001) (Table 1). BMI was significantly higher in both patient groups compared to the Healthy Group (*p* < 0.001), with no significant changes between baseline and follow‐up. The differences pre‐post treatments showed small effect sizes (Cohen's *d* < 0.3), apart from the high effect in TFA and MPTA (Cohen's *d* = 2.51 and −2.43). In addition, the VAS score significantly improved post‐treatment in the Surgical Group (from 6.0 ± 2.1 to 1.6 ± 1.9, *p* < 0.001), while it did not significantly change in the Conservative Group (from 4.0 ± 2.1 to 2.9 ± 2.3, *p* = 0.063) (Table 1).

Regarding spatio‐temporal parameters, the Surgical Group exhibited significantly lower speed, stride length, cadence and stance time during walking, compared to the Healthy Group at both baseline and follow‐up (*p* < 0.005), with no significant improvements after surgery (Table 2). A less marked trend was observed in the Conservative Group. No significant differences in spatio‐temporal parameters were found for stair ascent and stair descent across any condition in either group, except for cadence between baseline and healthy in the Surgical Group (Table 2). The differences pre‐ and post‐treatments showed small‐to‐moderate effect sizes (Cohen's *d* < 0.6), confirming that no clinically meaningful differences occurred for those parameters.

**Table 2:** Spatio‐temporal parameters (baseline, follow‐up and healthy) for the Surgical and Conservative groups.

Surgical Group							
	Baseline	Follow‐up	Healthy	Baseline vs. follow‐up	Baseline vs. healthy	Follow‐up vs. healthy	Effect size *–* baseline vs. follow‐up
	Mean (std)	Mean (std)	Mean (std)	*p*	*p*	*p*	Cohen's *d*
Speed (m/s)							
Walking	1.07 (0.14)	1.04 (0.16)	1.25 (0.11)	0.912	**<0.001**	**<0.001**	0.20
Stair ascent	0.40 (0.06)	0.44 (0.07)	0.45 (0.05)	0.368	0.050	0.579	−0.61
Stair descent	0.47 (0.10)	0.50 (0.11)	0.53 (0.06)	0.506	0.062	0.489	−0.29
Stride length (%Height)							
Walking	70.6 (5.8)	71.2 (7.1)	78.5 (4.3)	0.914	**<0.001**	**0.001**	−0.09
Stair ascent	40.3 (1.6)	41.1 (2.7)	41.5 (2.2)	0.511	0.218	0.841	−0.36
Stair descent	41.4 (2.6)	41.1 (3)	40.6 (3.3)	0.991	0.344	0.414	0.11
Cadence (stride/min)							
Walking	52.6 (3.2)	51.1 (3.4)	55.9 (2.8)	0.346	**0.005**	**<0.001**	0.45
Stair ascent	34.7 (4.9)	36.8 (5.1)	38.3 (4.1)	0.419	**0.046**	0.501	−0.42
Stair descent	39.0 (5.8)	41.7 (6.4)	45.6 (3.7)	0.273	**0.002**	0.133	−0.44
Stance time (%)							
Walking	61.9 (1.7)	61.5 (1.7)	59.3 (1.1)	0.603	**<0.001**	**<0.001**	0.24
Stair ascent	63.7 (1.7)	62.8 (1.7)	63.0 (2.6)	0.368	0.467	0.984	0.53
Stair descent	66.3 (3.2)	64.3 (3.7)	64.9 (2.7)	0.112	0.403	0.754	0.58

Conservative Group							
	Baseline	Follow‐up	Healthy	Baseline vs. follow‐up	Baseline vs. healthy	Follow‐up vs. healthy	Effect size – baseline vs. follow‐up
	Mean (std)	Mean (std)	Mean (std)	*p*	*p*	*p*	Cohen's *d*
Speed (m/s)							
Walking	1.19 (0.10)	1.16 (0.11)	1.25 (0.11)	0.716	0.185	**0.034**	0.29
Stair ascent	0.43 (0.07)	0.45 (0.05)	0.45 (0.05)	0.232	0.312	0.996	−0.33
Stair descent	0.49 (0.10)	0.53 (0.07)	0.53 (0.06)	0.102	0.269	0.924	−0.46
Stride length (%Height)							
Walking	74.5 (3.7)	73.6 (4.6)	78.5 (4.3)	0.840	**0.012**	**0.002**	0.22
Stair ascent	40.3 (2.2)	40.4 (2.3)	41.5 (2.2)	0.979	0.336	0.437	−0.04
Stair descent	39.8 (3.2)	40.4 (3.4)	40.6 (3.3)	0.729	0.955	0.905	−0.18
Cadence (stride/min)							
Walking	54.5 (3.7)	53.7 (4.0)	55.9 (2.8)	0.754	0.241	0.058	0.21
Stair ascent	36.3 (5.5)	38.0 (3.4)	38.3 (4.1)	0.223	0.202	0.988	−0.37
Stair descent	42.4 (7.3)	44.5 (5.2)	45.6 (3.7)	0.246	0.082	0.801	−0.33
Stance time (%)							
Walking	61.1 (1.3)	60.7 (1.6)	59.3 (1.1)	0.656	**<0.001**	**0.002**	0.27
Stair ascent	63.0 (1.7)	62.4 (2.1)	63.0 (2.6)	0.528	0.971	0.713	0.31
Stair descent	66.1 (2.3)	65.1 (2.1)	64.9 (2.7)	0.357	0.377	0.999	0.45

*Note*: Bold *p* values indicate statistically significant difference.

### Kinematics during motor activities

**Figure 2:**
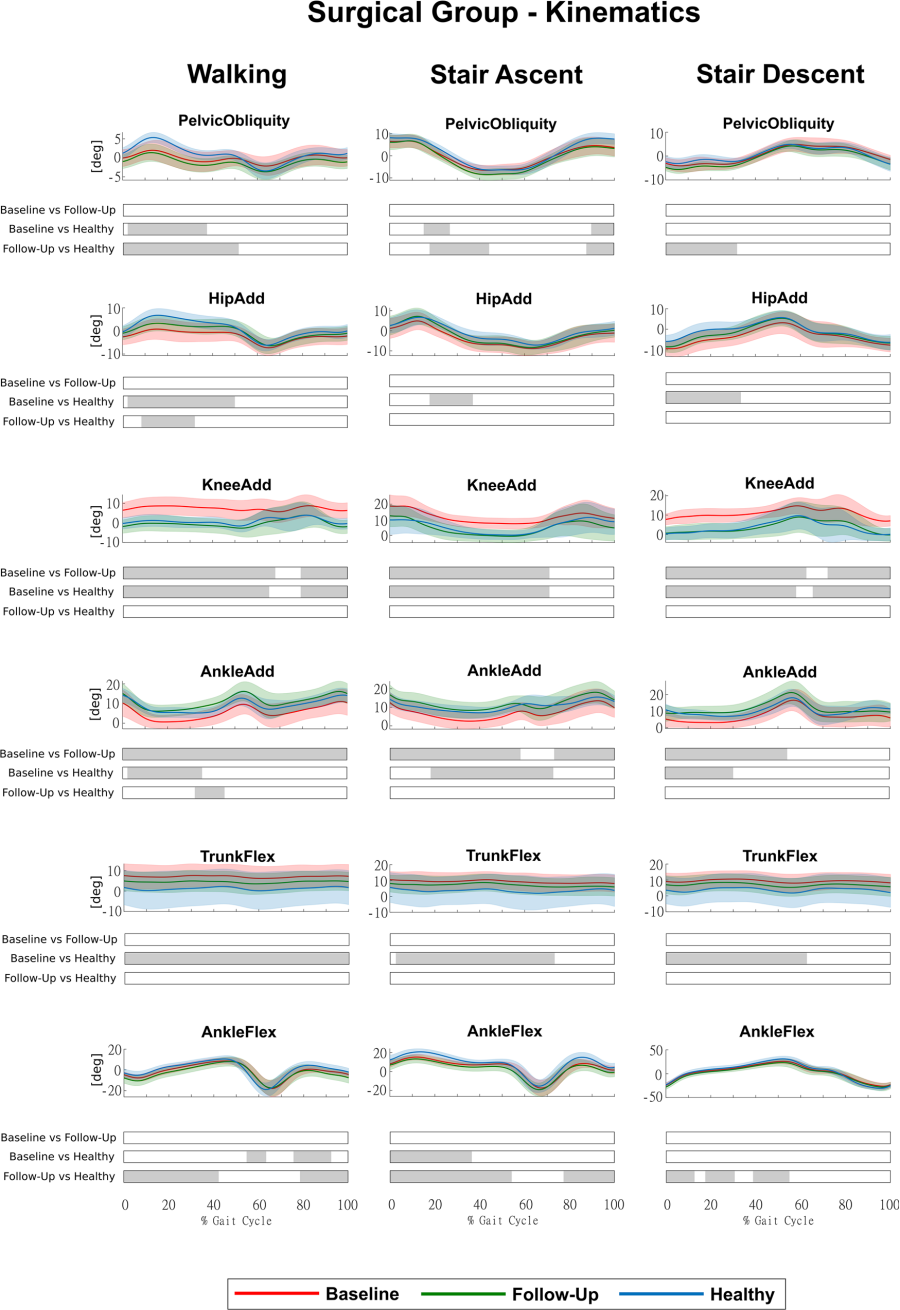
Kinematics of the Surgical Group for the three conditions (baseline, follow‐up and healthy) during walking, stair ascent and stair descent (mean ± std). Time intervals during the movement cycle with statistically significant differences between groups (post hoc SPM *t* tests) are reported as grey bars below each subplot. SPM, statistical parametric mapping.

**Figure 3:**
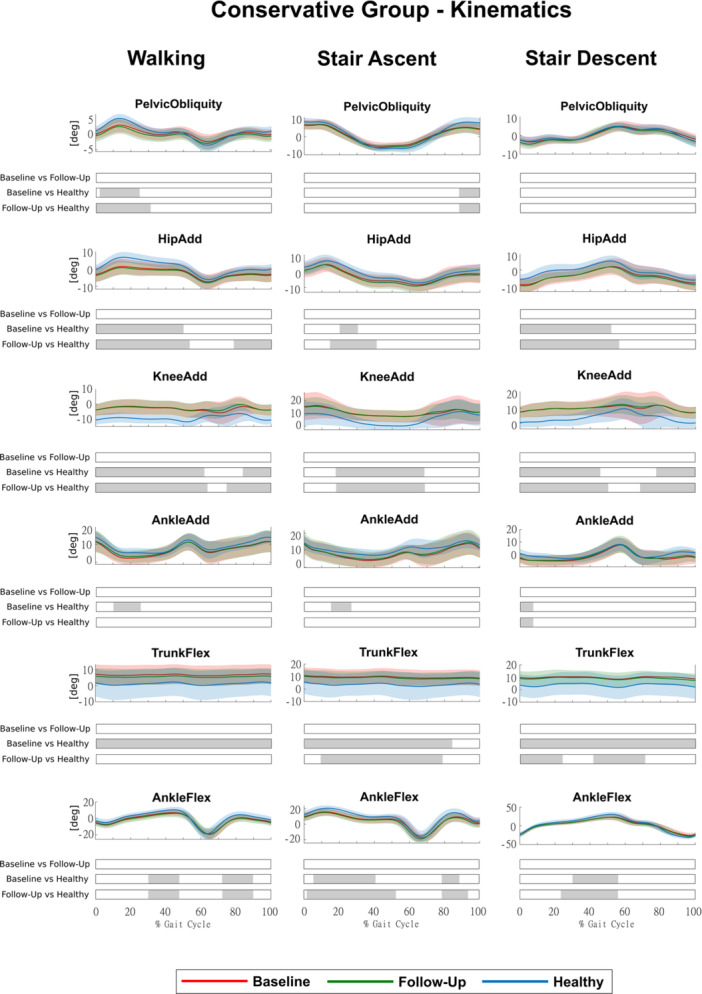
Kinematics of the Conservative Group for the three conditions (baseline, follow‐up and healthy) during walking, stair ascent and stair descent (mean ± std). Time intervals during the movement cycle with statistically significant differences between groups (post hoc SPM *t* tests) are reported as grey bars below each subplot. SPM, statistical parametric mapping.

HTO led to significant improvements at follow‐up (Surgical Group), in several joint rotations that were altered at baseline in all motor tasks (Figure 2), whereas conservative treatments did not, as no significant changes were observed at follow‐up (Conservative Group) (Figure 3).

In the Surgical Group, knee and ankle adduction, which were markedly altered at baseline in all motor activities, were fully restored, with significant differences between baseline and follow‐up for at least 60% of the movement cycles and no significant differences between follow‐up and Healthy Group, except for a 22% portion of the gait cycle in ankle adduction during walking (Figure 2). Hip adduction and trunk flexion showed partial improvements, trending towards healthy values, although no significant differences were detected between baseline and follow‐up. Conversely, pelvic obliquity and ankle flexion exhibited persistent alterations, as differences with the Healthy Group remained significant at follow‐up, with ankle flexion even showing a tendency towards greater deviations (Figure 2). In the Conservative Group, we found no significant differences in any joint rotations between baseline and follow‐up (Figure 3). In addition, we found no significant differences between the Surgical and Conservative groups at baseline for any kinematic parameters. However, at follow‐up, pelvic obliquity, hip, knee and ankle adduction showed significant differences between groups, confirming greater improvements in the Surgical Group (Figure S2).

### Kinetics during motor activities

**Figure 4:**
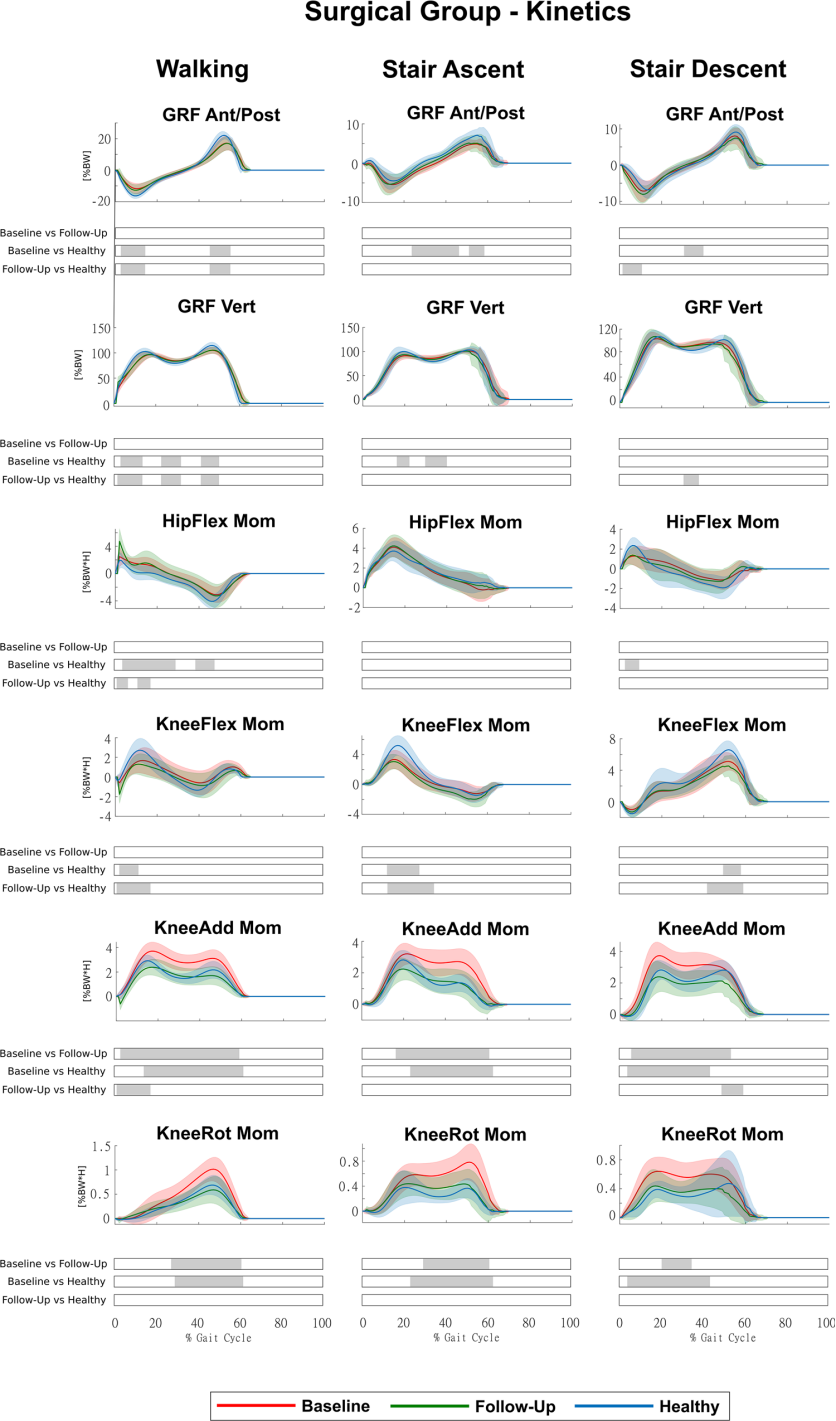
Kinetics of the Surgical Group for the three conditions (baseline, follow‐up and healthy) during walking, stair ascent and stair descent (mean ± std). Time intervals during the movement cycle with statistically significant differences between groups (post hoc SPM *t* tests) are reported as grey bars below each subplot. SPM, statistical parametric mapping.

**Figure 5:**
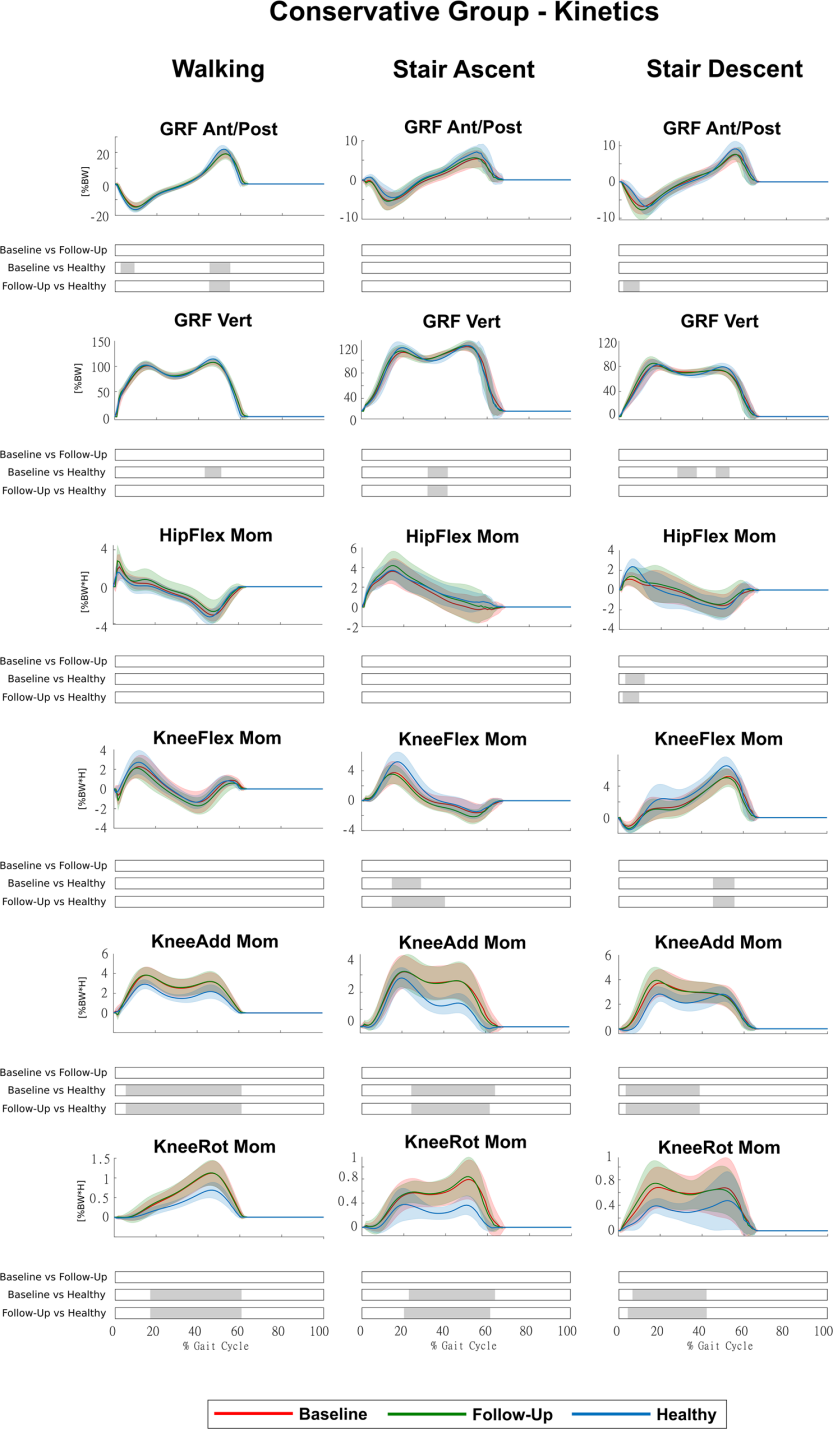
Kinetics of the Conservative Group for the three conditions (baseline, follow‐up and healthy) during walking, stair ascent and stair descent (mean ± std). Time intervals during the movement cycle with statistically significant differences between groups (post hoc SPM *t* tests) are reported as grey bars below each subplot. SPM, statistical parametric mapping.

HTO led to significant improvements at follow‐up (Surgical Group) in several joint moments that were altered at baseline (Figure 4), whereas conservative treatments did not, as no significant changes were observed at follow‐up (Conservative Group) (Figure 5).

In the Surgical Group, KAM and KRM, which were markedly altered at baseline in all motor activities, were fully restored, with significant differences between baseline and follow‐up for most of the movement cycles. We found no significant differences between follow‐up and healthy, with a small portion in KAM even significantly lower than healthy during walking (Figure 4). Antero‐posterior and vertical ground reaction forces, as well as hip and KFMs, showed persistent alterations, as the differences with the healthy remained significant at follow‐up, although for small portions of the movement cycles (Figure 4). In the Conservative Group, we found no significant differences in any kinetic parameters between baseline and follow‐up (Figure 5). In addition, we found no significant differences between the Surgical and Conservative groups at baseline for any kinetic parameters. However, at follow‐up, KAM and KRM showed significant differences between groups, confirming greater improvements in the Surgical Group (Figure S3).

## DISCUSSION

In this study, we found that HTO effectively restores specific biomechanical alterations during walking, stair ascent and stair descent, whereas conservative treatments did not lead to significant improvements. Therefore, our hypothesis that patients undergoing HTO would demonstrate greater improvements compared to those receiving conservative treatment was confirmed. However, a few persistent motor function deviations were still observed after HTO, suggesting that full biomechanical recovery may require additional treatments, such as rehabilitation strategies that target both postural control and dynamic load redistribution.

Our results confirmed that HTO effectively corrects knee and ankle adduction angles, restoring them to those observed in healthy individuals (Figure 2). In addition, HTO effectively reduces and restores KAM and KRM, shifting the load distribution away from the medial compartment (Figure 4). These findings align with previous studies [7, 18], which demonstrated that HTO successfully redistributes knee joint loads and improves lower limb alignment. Our results are also consistent with studies that highlight HTO as a key intervention for unloading the medial knee joint [1, 24, 25]. Interestingly, our study found that KAM around the first peak during walking was even significantly lower than in healthy individuals. While this suggests successful offloading, it may also indicate a potential overcorrection, where patients adopt a compensatory strategy that excessively reduces medial knee loading, although the clinical significance of this overcorrection remains speculative. Recent research [7] also reported that some post‐HTO patients exhibit an excessive shift in knee joint moments, which could have unintended consequences on lateral knee compartment stress. Although mechanical realignment is a key factor in restoring joint loading, individual patient characteristics may also influence post‐operative gait adaptations. Higher BMI has been associated with altered joint mechanics and loading patterns in knee OA [9, 10], while persistent compensatory strategies such as pelvic obliquity or trunk adaptations may reflect chronic biomechanical habits [27]. These aspects, not directly addressed in this study, warrant further investigation in future work.

Despite these marked improvements, hip adduction and trunk flexion did not show significant changes post‐surgery, suggesting that proximal compensatory strategies persist even after knee realignment (Figure 2). This is consistent with previous research that found that HTO improves frontal plane knee mechanics but does not fully restore proximal kinematics [33]. Also, pelvic obliquity and ankle flexion remained significantly altered at follow‐up, with ankle flexion even showing a tendency toward greater deviations (Figure 2). This could be attributed to persistent neuromuscular imbalances, or modifications in gait strategy after surgery [27], potentially suggesting a need for additional rehabilitation strategies beyond restoring mechanical TFA. In addition, antero‐posterior and vertical ground reaction forces, as well as hip and KFMs, remained altered, although differences from healthy controls were limited to small portions of the movement cycles (Figure 4). Our findings about the knee moments not restored on the sagittal plane agree with most studies on HTO patients [2, 7, 16, 33]. This suggests that HTO does not fully normalize the dynamic loading response of the lower limb in about one year after surgery, potentially due to residual neuromuscular adaptations or altered postural control strategies [9, 10].

Regarding spatio‐temporal parameters, we found that HTO did not lead to improvements in walking speed and stride length, but stair locomotion performance was comparable to healthy conditions (Table 2). Walking speed and stride length remained significantly lower than in healthy individuals, with no significant changes between baseline and follow‐up. Similarly, stair ascent and descent speeds did not improve post‐surgery, indicating that HTO successfully corrects knee alignment but does not necessarily enhance overall gait performance. These findings align with previous studies [2, 20], which reported that HTO effectively modifies knee joint mechanics but does not always translate into improved walking speed or functional mobility. One possible explanation is that neuromuscular control deficits persist even after mechanical realignment, leading to a prolonged adaptation phase before functional recovery is achieved [15]. Additionally, fear of instability or pain‐related movement modifications could influence spatio‐temporal parameters [8].

A key strength of our study is its prospective randomized case‐control design, which directly compares the effects of HTO with a non‐surgical conservative treatment. To our knowledge, this is the first study to employ such a methodology in the context of patients with knee OA with varus malalignment eligible for HTO. While previous studies have assessed post‐HTO biomechanical changes [7, 33], they lacked a direct control group receiving non‐surgical management. The inclusion of a conservative treatment group allowed us to distinguish whether observed biomechanical improvements result from surgical intervention itself or from general adaptations over time. Our findings clearly indicate that HTO was necessary to achieve significant biomechanical improvements, as no relevant changes were observed in the Conservative Group at follow‐up. In addition, the absence of significant differences in motor function between the two groups at baseline confirms that both cohorts were biomechanically comparable before treatment. The significant between‐group differences observed at follow‐up (see Supplementary Material) further support the conclusion that the improvements in the Surgical Group can be attributed to the effects of HTO rather than time alone or baseline heterogeneity. Also, knee pain (see VAS score) was markedly improved after HTO, while it did not significantly change after non‐surgical treatment.

While the HTO procedure effectively corrects mechanical alignment and most of joint loading and kinematic parameters, persistent pelvic obliquity, ankle flexion deficits and reduced walking speed suggest post‐surgical rehabilitation programmes that could be focused on strengthening exercises for hip abductors and core stabilizers [3, 31], and gait retraining interventions, including proprioceptive exercises and ankle mobility work, to address residual deficits in ankle flexion and foot mechanics [28].

This study has some limitations that should be addressed in future research. First, the mean follow‐up period was 19 months, which may not be sufficient to capture long‐term movement adaptations. Future studies should evaluate whether still altered biomechanical parameters improve, or are maintained, over longer recovery periods. Second, surface EMG could provide additional specific insights into muscular compensations that persist after HTO, particularly in the hip and ankle joints. Third, while our findings suggest that some patients may exhibit overcorrection, future research should investigate optimal correction thresholds to balance offloading the medial compartment without excessive reduction of KAM. Fourth, the gender imbalance of our sample reflects the demographic distribution of patients eligible for HTO in our clinical setting. We found that gender differences in gait patterns did not bias the overall study conclusions, as no significant differences were observed between male and female participants at baseline for the key gait parameters (Figure S1). Finally, the Healthy Group was younger and structurally different from the patient groups, and no radiographic data were available for these subjects. However, this group was included to provide a reference for normal motor function rather than for direct anatomical comparison.

The observed gait adaptations reflect important outcomes at a mid‐term follow‐up, and some residual deviations, especially in sagittal plane mechanics, may further evolve over time, as suggested by previous studies reporting delayed recovery of KFMs [2, 7, 16, 33]. While all patients underwent standardized rehabilitation, individual variability in recovery might also be influenced by adherence or neuromuscular adaptation, which were not specifically assessed in this study.

## CONCLUSION

HTO effectively restores joint alignment and major motor function parameters during walking and stair tasks in patients with knee OA and varus knee, confirming its role in redistributing medial knee loads, whereas non‐surgical conservative treatments do not improve motor function. However, persistent alterations in a few kinematic and kinetic parameters suggest that certain biomechanical adaptations may remain after surgical correction. These findings provide further insights into the functional impact of HTO and its role in improving motor function parameters.

## AUTHOR CONTRIBUTIONS

**Giordano Valente**: Conceptualization; formal analysis; investigation; methodology; software; supervision; visualization; writing—original draft; writing—review and editing. **Giulia Grenno**: Formal analysis; methodology; software; writing—review and editing. **Giacomo Dal Fabbro**: Conceptualization; data curation; investigation; project administration; writing—review and editing. **Alberto Grassi**: Investigation; writing—review and editing. **Alberto Leardini**: Data curation; writing—review and editing. **Lisa Berti**: Supervision; writing—review and editing. **Stefano Zaffagnini**: Conceptualization; funding acquisition; project administration; supervision, writing—review and editing. **Fulvia Taddei**: Conceptualization; funding acquisition; project administration; supervision; writing—review and editing.

## CONFLICT OF INTEREST STATEMENT

The authors declare no conflicts of interest.

## ETHICS STATEMENT

Approval for this work was granted by the local Ethics Committee: CE‐AVEC 440/2019/Sper/IOR. Written informed consent was obtained from each participant prior to data collection.

## Supporting information

Figure S1. Comparison of kinematics and kinetics parameters between male and female participants at baseline (mean ± std). Only the parameters showing statistically significant differences between groups in some time intervals (post‐hoc SPM t‐tests) were reported. All the other parameters did not show statistically significant differences.

Figure S2. Comparison of kinematics parameters at follow‐up between the Surgical and Conservative groups during walking, stair ascent and stair descent (mean ± std). Time intervals during the movement cycle with statistically significant differences between groups (post‐hoc SPM t‐tests) are reported as gray bars below each subplot.

Figure S3. Comparison of kinetics parameters at follow‐up between the Surgical and Conservative groups during walking, stair ascent and stair descent (mean ± std). Time intervals during the movement cycle with statistically significant differences between groups (post‐hoc SPM t‐tests) are reported as gray bars below each subplot.

Table S1. Patients' characteristics (Surgical vs Conservative Group) at Baseline and Follow‐up. Bold p‐values indicate statistically significant differences.

## Data Availability

Data used in the study are available upon appropriate request.
