# Supplementary material for: High tibial osteotomy effectively restores motor function during daily activities in patients with knee osteoarthritis and varus deformity
Source: J Exp Orthop. 2025 Sep 15;12(3):e70410. doi: 10.1002/jeo2.70410 (PMC12435303; doi:10.1002/jeo2.70410)
Supplement: Supplementary file 1 — Figure S1. Comparison of kinematics and kinetics parameters between male and female participants at baseline (mean ± std). Only the parameters showing statistically significant differences between groups in some time intervals (post‐hoc SPM t‐tests) were reported. All the other parameters did not show statistically significant differences. [file JEO2-12-e70410-s003.docx]

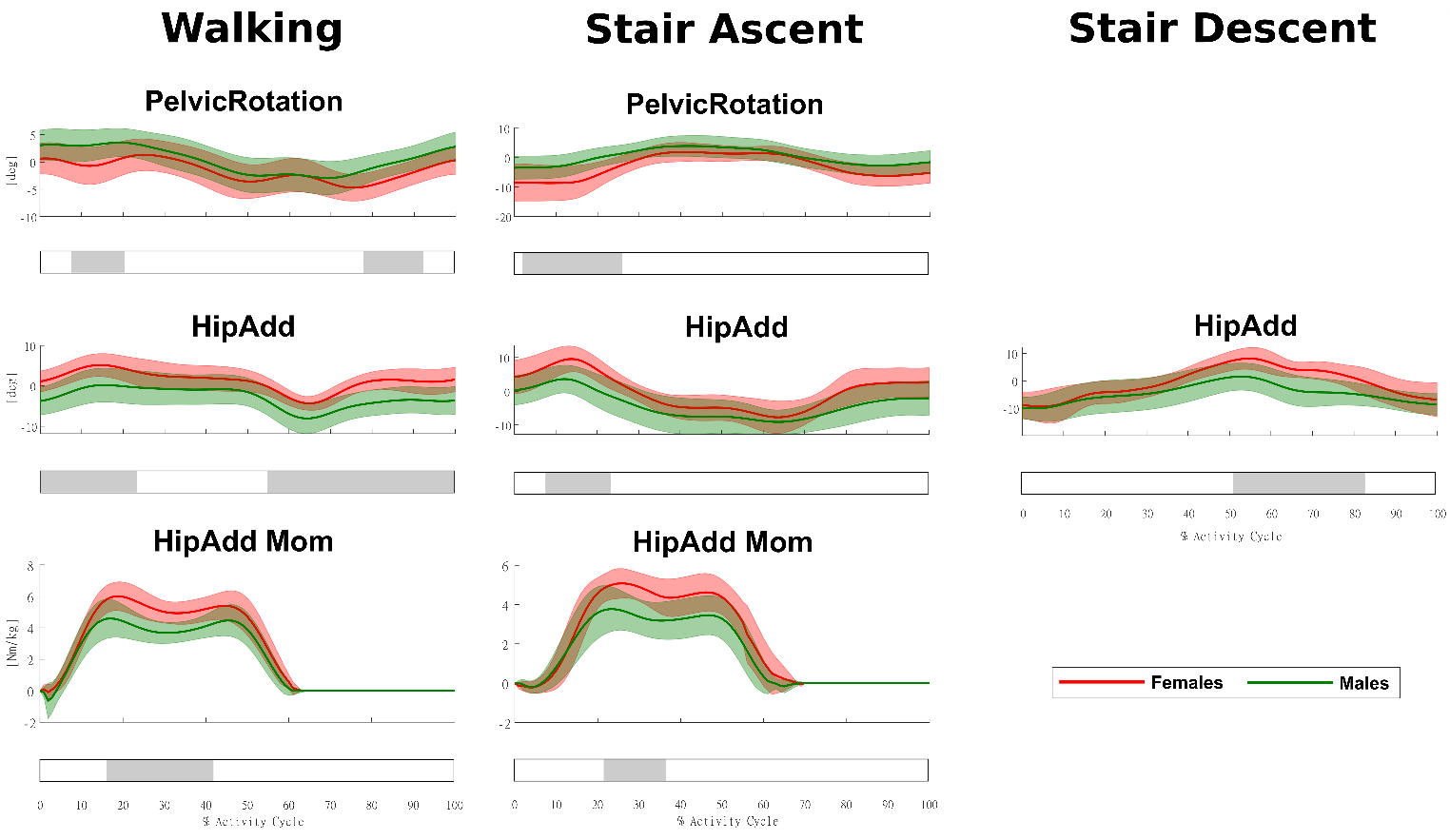


Figure S1. Comparison of kinematics and kinetics parameters of pre-treatment patients between females and males. Statistical Parametric Mapping t-tests results are shown only for the variables where some significant differences occurred during the activity cycles, revealing no significant differences for most variables and suggesting that gender differences were minimal.
