# Supplementary material for: High tibial osteotomy effectively restores motor function during daily activities in patients with knee osteoarthritis and varus deformity
Source: J Exp Orthop. 2025 Sep 15;12(3):e70410. doi: 10.1002/jeo2.70410 (PMC12435303; doi:10.1002/jeo2.70410)
Supplement: Supplementary file 2 — Figure S2. Comparison of kinematics parameters at follow‐up between the Surgical and Conservative groups during walking, stair ascent and stair descent (mean ± std). Time intervals during the movement cycle with statistically significant differences between groups (post‐hoc SPM t‐tests) are reported as gray bars below each subplot. [file JEO2-12-e70410-s001.docx]

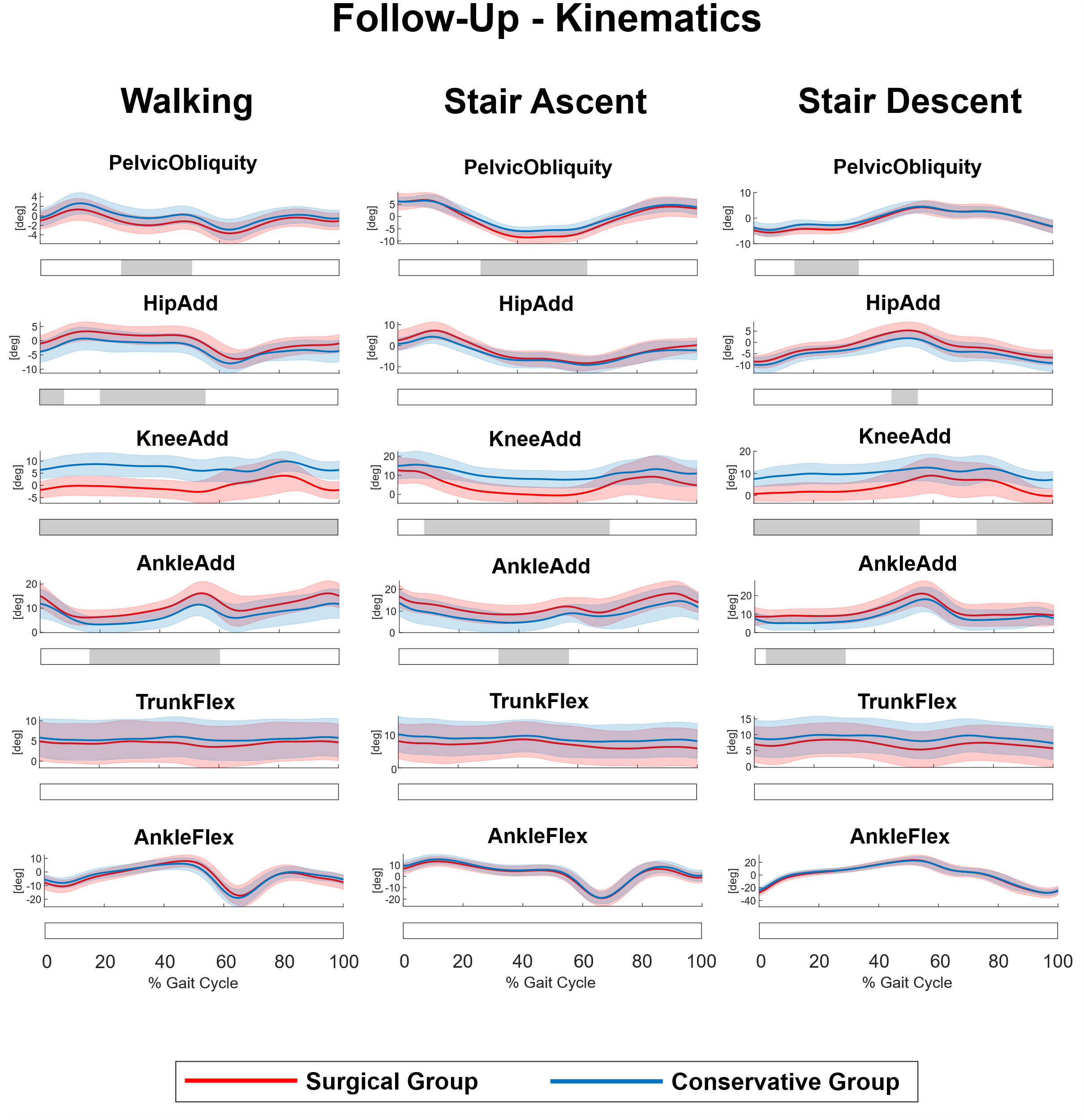


Figure S2. Comparison of kinematics parameters at follow-up between the Surgical and Conservative groups during walking, stair ascent and stair descent (mean ± std). Time intervals during the movement cycle with statistically significant differences between groups (post-hoc SPM t-tests) are reported as gray bars below each subplot.
