# Supplementary material for: High tibial osteotomy effectively restores motor function during daily activities in patients with knee osteoarthritis and varus deformity
Source: J Exp Orthop. 2025 Sep 15;12(3):e70410. doi: 10.1002/jeo2.70410 (PMC12435303; doi:10.1002/jeo2.70410)
Supplement: Supplementary file 4 — Table S1. Patients' characteristics (Surgical vs Conservative Group) at Baseline and Follow‐up. Bold p‐values indicate statistically significant differences. [file JEO2-12-e70410-s002.docx]

| **Table S1. Patients’ characteristics (Surgical vs Conservative Group) at Baseline and Follow-up. Bold p-values indicate statistically significant differences.** | | | |
| --- | --- | --- | --- |
| **Patients at Baseline** | | | |
|  | **Surgical Group** | **Conservative Group** | **Surgical vs Conservative** |
|  | mean (std) | mean (std) | p-value |
| **Number of participants** | 25 | 24 | - |
| **Age (yrs)** | 54.3 (6.8) | 51.6 (10.0) | 0.516 |
| **Gender (F/M)** | 7F / 18M | 2F / 22M | - |
| **Body Mass Index (kg/cm^2^)** | 26.9 (4.0) | 25.6 (3.1) | 0.308 |
| **Tibiofemoral angle (deg)** | 8.3 (3.3) | 7.7 (4.0) | 0.352 |
| **Patients at Follow-up** | | | |
|  | **Surgical Group** | **Conservative Group** | **Surgical vs Conservative** |
|  | mean (std) | mean (std) | p-value |
| **Number of participants** | 25 | 24 | - |
| **Age (yrs)** | 55.8 (6.7) | 53.0 (9.9) | 0.395 |
| **Gender (F/M)** | 7F / 18M | 2F / 22M | - |
| **Body Mass Index (kg/cm^2^)** | 28.0 (4.2) | 25.9 (3.4) | 0.168 |
| **Tibiofemoral angle (deg)** | 0.9 (2.4) | 7.6 (4.4) | **<0.001** |
|  |  |  |  |
